# Unveiling orphan receptor-like kinases in plants: novel client discovery using high-confidence library predictions in the Kinase–Client (KiC) assay

**DOI:** 10.3389/fpls.2024.1372361

**Published:** 2024-04-03

**Authors:** Gabriel Lemes Jorge, Daewon Kim, Chunhui Xu, Sung-Hwan Cho, Lingtao Su, Dong Xu, Laura E. Bartley, Gary Stacey, Jay J. Thelen

**Affiliations:** ^1^ Division of Biochemistry, C.S. Bond Life Sciences Center, University of Missouri, Columbia, MO, United States; ^2^ Division of Plant Science & Technology, C.S. Bond Life Sciences Center, University of Missouri, Columbia, MO, United States; ^3^ Institute for Data Science and Informatics, C.S. Bond Life Sciences Center, University of Missouri, Columbia, MO, United States; ^4^ Department of Electrical Engineering and Computer Science, C.S. Bond Life Sciences Center, University of Missouri, Columbia, MO, United States; ^5^ Shandong University of Science and Technology, Qingdao, Shandong, China; ^6^ Institute of Biological Chemistry, Washington State University, Pullman, WA, United States

**Keywords:** protein kinase, *in vitro* screening, deep learning, phosphopeptides, P2K1, extracellular ATP

## Abstract

Plants are remarkable in their ability to adapt to changing environments, with receptor-like kinases (RLKs) playing a pivotal role in perceiving and transmitting environmental cues into cellular responses. Despite extensive research on RLKs from the plant kingdom, the function and activity of many kinases, i.e., their substrates or “clients”, remain uncharted. To validate a novel client prediction workflow and learn more about an important RLK, this study focuses on P2K1 (DORN1), which acts as a receptor for extracellular ATP (eATP), playing a crucial role in plant stress resistance and immunity. We designed a Kinase-Client (KiC) assay library of 225 synthetic peptides, incorporating previously identified P2K phosphorylated peptides and novel predictions from a deep-learning phosphorylation site prediction model (MUsite) and a trained hidden Markov model (HMM) based tool, HMMER. Screening the library against purified P2K1 cytosolic domain (CD), we identified 46 putative substrates, including 34 novel clients, 27 of which may be novel peptides, not previously identified experimentally. Gene Ontology (GO) analysis among phosphopeptide candidates revealed proteins associated with important biological processes in metabolism, structure development, and response to stress, as well as molecular functions of kinase activity, catalytic activity, and transferase activity. We offer selection criteria for efficient further *in vivo* experiments to confirm these discoveries. This approach not only expands our knowledge of P2K1’s substrates and functions but also highlights effective prediction algorithms for identifying additional potential substrates. Overall, the results support use of the KiC assay as a valuable tool in unraveling the complexities of plant phosphorylation and provide a foundation for predicting the phosphorylation landscape of plant species based on peptide library results.

## Introduction

Plants, as sessile organisms, exhibit a remarkable ability to adapt to their changing environments. A key to their adaptability is the intricate network of signaling pathways that underlie a wide range of physiological responses. Among the numerous signaling molecules and receptors involved, membrane-spanning receptor-like kinases (RLKs) stand out as integral components in perceiving and transducing environmental cues into cellular responses due to their greatly expanded numbers in plant genomes relative to those of animals (Shiu and Bleecker, 2001; Choi et al., 2014). Numerous RLKs have been found to have critical functions in innate (cellular) immunity and coordination of the complex processes of multicellular development (Lin et al., 2013; Tang et al., 2017). However, the function, including receptor ligands and downstream signaling clients, for most RLKs remains uncharted within the realm of plant biology.

In plants and animals, extracellular ATP (eATP) is released during tissue damage or in response to specific elicitation, including pathogens (Burnstock and Knight, 2004), and acts as a bridge between the extracellular environment and intracellular signaling pathways. By binding to various ligands, including peptides, proteins, and small molecules, RLKs can activate cascades of downstream events that are essential for growth, development, and adaptation to environmental changes (Choi et al., 2014). A member of the lectin-RLK subfamily, DORN1 (Does not Respond to Nucleotides 1), also known as P2K1 to align with animal purino-receptor nomenclature, was first identified in plants (Choi et al., 2014). This key receptor recognizes eATP and plays a variety of roles in plant stress resistance and is recognized as the primary eATP receptor in Arabidopsis (Choi et al., 2014). In addition, P2K1 induces an innate immunity response against the oomycete pathogens, *Phytophthora brassicae* (Bouwmeester et al., 2011), *Phytophthora infestans* (Bouwmeester et al., 2014), and bacterial pathogen *Pseudomonas syringae* DC3000 (Balagué et al., 2017). Another RLK, P2K2 may form a heteromeric complex with P2K1, especially under higher stress conditions where its expression is induced, potentially enhancing the specificity and intensity of eATP cellular responses (Pham et al., 2020).

One strategy to uncover the functions of RLKs is phosphoproteomic analysis, which catalogs the *in vivo* phosphorylation of proteins in response to specific stimuli. However, distinguishing direct and indirect phosphorylation events makes it difficult to pinpoint RLK-specific responses accurately. The Kinase–Client (KiC) assay addresses the challenge of identifying likely clients for RLKs in a more direct manner. Purified kinases are exposed to a synthetic peptide library and resulting phosphorylated peptides detected by liquid chromatography (LC)-tandem MS (MS/MS) analysis (Huang and Thelen, 2012). By tracking spectral counts of phosphorylated and unphosphorylated peptides, this approach directly quantifies kinase activity and provides superior signal-to-noise ratios, sensitivity, and specificity compared to other large-scale methods for studying kinase substrates (Xue and Tao, 2013). This method assumes that the amino acid sequence surrounding phosphorylation sites plays a crucial role in determining the substrate specificity of protein kinases (Huang and Thelen, 2012). Moreover, it is a valuable technique to discriminate phosphorylation site ambiguity on phosphopeptides, especially in cases where multiple serine, threonine, or tyrosine (S/T/Y) residues are present in a single peptide (Ahsan et al., 2013). Mass spectrometry has been widely used to screen kinase inhibitors and monitor kinase activity because of its sensitive and accurate nature (Gao and Leary, 2003; Zhang et al., 2009). Also, MS-based approaches have been shown to be a useful tool in the study of kinase-client protein interactions, and the usage of spectral counting has been previously utilized to quantify phosphopeptides by establishing the *in vitro* kinetic constants for kinases (Huang et al., 2010).

Over the last few years, several phosphopeptide candidates identified by KiC assay screening with P2K1 have been experimentally confirmed. In plant innate immunity, the protein modification S-acylation of the P2K1 receptor by Arabidopsis PAT5 and PAT9 affects the temporal dynamics of its activity, influencing the immune response by regulating receptor phosphorylation and degradation in response to external stimuli (Chen et al., 2021). Another P2K1 kinase client identified through KiC Assay screening, revealed that eATP also elicits P2K1-mediated RBOHD phosphorylation to regulate stomatal aperture with important implications for regulating plant photosynthesis, water homeostasis, pathogen resistance, and ultimately yield (Chen et al., 2017).

Previous KiC assay peptide libraries were designed based on experimentally observed phosphopeptides (Ahsan et al., 2013), as this is a rational approach for designing a library given the significant expense of synthetic peptides. However, experimental phosphopeptide catalogs for any plant species are likely incomplete for both technical and biological reasons, e.g. transient clients that do not accumulate *in vivo*. Advances in *in silico* prediction methods can be used to predict additional kinase clients beyond the limitations of current datasets and cost-limited peptide libraries. Various methodologies, including support vector machines (Kim et al., 2004), neural networks (Blom et al., 2004), and conditional random field Models (Dang et al., 2008), coupled with bootstrap aggregation procedures and integrating sequence cluster information can be used to predict phosphorylation sites (Gao et al., 2010). In the training, the combination of protein sequences and functional features (Song et al., 2017), high-throughput proteomic-scale predictions (Li et al., 2018), position-weight determination or scoring-matrix optimization (Wang et al., 2020), and machine learning-based approaches (Ma et al., 2023) have been employed for predicting kinase-specific substrates and associated phosphorylation sites. MUsite is another tool that employs a machine-learning approach and integrates diverse factors, including local sequence similarities, protein disorder scores, amino acid frequencies, and is trained on phosphoproteomics data from multiple organisms (Gao et al., 2010; Yao et al., 2012). These strategies make it possible to choose among peptide candidates, which saves money and time during the experimental phase. Additionally, the diversity of the library can be increased by the addition of *in silico* predictions. Furthermore, prediction methods typically provide confidence scores, ranking peptide candidates and increasing the chances of finding new and physiologically meaningful phosphorylation events.

Here, we present an innovative KiC assay screen that underscores the significance of prediction tools. By leveraging HMMER (Potter et al., 2018) and MUsite (Gao et al., 2010; Yao et al., 2012), combined with a robust foundation of previously experimentally validated phosphopeptides, we generated a 225-peptide library for *in vitro* P2K screening. Through the implementation of an optimized screening workflow utilizing a purified P2K1-CD enzyme, we successfully identified 46 substrates for P2K1, including 34 novel clients, 27 of which are not present in a large experimental database. This not only showcases the efficacy of prediction methods but also highlights crucial steps for further phosphopeptide candidate selection criteria.

## Materials and methods

### Kinase assay and synthetic peptide library preparation

**Figure 1:**
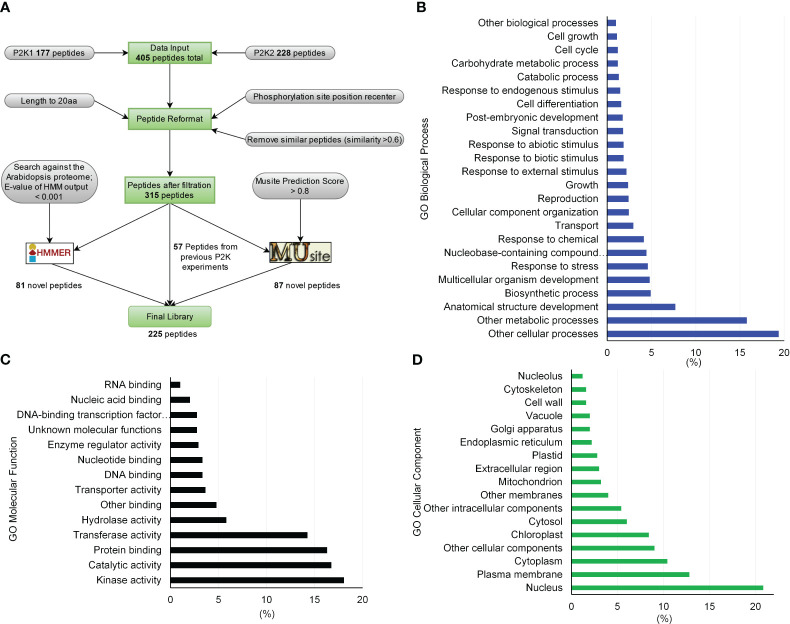
Peptide library generation pipeline for substrates of receptor-like kinases in plants and library gene ontology (GO) enrichment characteristics. **(A)** By formatting and filtering peptides from the previously identified peptide substrates, only peptides with phosphorylation sites at least 5 amino acids away from either end were kept, and similar or duplicated peptides were removed. Proteins containing these peptides were identified and peptides were kept with a length of 20 aa by either truncating or expanding the original peptides. Together, 315 peptides were used to build sequence profiles to train HMMER and MUsite. The trained models were searched against the whole Arabidopsis proteome to predict new candidates for the substrates of P2Ks. In total, 225 high-confidence potential candidates were designed after all filtration criteria. **(B)** through **(D)** GO enrichment of the proteins corresponding to the 225-peptide library. Only gene ontology classes that had higher than 1% abundance among the 225-peptide library are shown for **(B)** GO Biological Process, **(C)** GO Molecular Function, and **(D)** GO Cellular Component.

We generated a peptide library of 225 peptides as candidate substrates for P2Ks from three sources – previous experiments, sequence homology based on a hidden Markov model (HMM), and a machine learning algorithm (**Figure 1A**). First, the interacting peptides with P2Ks on an initial KiC assay library were obtained and peptides with phosphorylation sites at least five amino acids away from either end were kept. This resulted in 405 peptides (177 and 228 from P2K1 and P2K2, respectively). Then similar or duplicated peptides (>0.6 sequence similarity) were removed, and 315 peptides in total remained. These peptides were reformatted into a 20-amino acid length, with phosphorylation sites located at the center. Furthermore, we used the 315 peptides to build profiles to train an HMM (Potter et al., 2018) and used the HMM to search the Arabidopsis proteome. The search yielded 81 candidate peptides with an E-value output < 0.001. (3) We also used the 315 peptides to train a deep-learning phosphorylation site prediction model MUsite (Gao et al., 2010; Yao et al., 2012) and used the model to search against the Arabidopsis proteome. These 87 possible candidate peptides were obtained with a prediction score > 0.8. In addition, we randomly selected 57 peptides out of the 315 peptides as positive controls to gauge the performance of the *in silico* predictions against previously identified phosphosites, providing insight into their accuracy. The final peptide library was composed of 225 peptides (81 + 87 + 57; **Supplementary Table 1**). Peptides were custom synthesized by GenScript using standard solid-phase peptide synthesis and purified by high-performance liquid chromatography.

**Figure 2:**
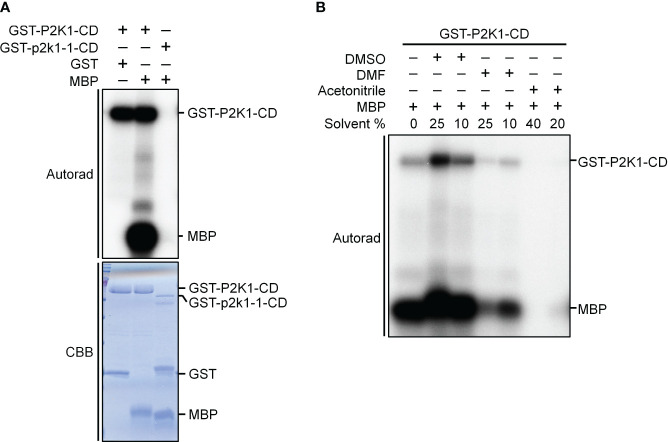
P2K1-CD shows auto- and trans-phosphorylation activities on myelin basic protein (MBP), and the phosphorylation activities are affected by solvents commonly used to solubilize synthetic peptides. **(A)** Incubation of MBP with GST-P2K1- cytosolic domain (CD), GST-p2k1-1-CD (kinase dead), or GST in an *in vitro* kinase assay. Autophosphorylation and trans-phosphorylation were measured by incorporation of γ-[32 P]-ATP. GST was used as a negative control. Protein content was visualized by Coomassie brilliant blue (CBB) staining. **(B)** Incubation of MBP protein with GST-P2K1-CD cytosolic domain in different solvents [DMSO (final 25%, 10%), DMF (final 25%, 10%), acetonitrile (final 40%, 20%)]. Autophosphorylation and trans-phosphorylation were measured upon addition of γ-[^32^ P]-ATP.

Kinase activity assays were conducted as previously described (Cho et al., 2022). Briefly, the cDNA of the P2K1 cytosolic domain (CD) and the kinase-dead version of GST-P2K1-CD (D572N; p2k1-1-CD) were ligated into pGEX-5X-1 resulting in N-terminal GST-tag fusion constructs (Choi et al., 2014). Kinase activity was measured by incubating 5 μg of purified GST-P2K1-CD and GST-p2k1-1-CD with 2 μg Myelin basic protein (MBP) as substrate in a 30 μL reaction [final conditions: 20 mM Tris-HCl pH 7.5, 10 mM MgCl_2_, 5 mM EGTA, 100 mM NaCl, and 1 mM DTT, 2 mM ATP, and 10 μCi radioactive [γ-^32^P] ATP, DMSO (final 25% or 10%), DMF (final 25% or 10%), acetonitrile (final 40% or 20%)] for 1.5 hours at 30°C. The kinase reactions were stopped by boiling with 5x SDS loading buffer for 5 minutes. After electrophoresis in 12% SDS-PAGE, the gel was exposed for 12 hours for autoradiography. The phosphorylation images were obtained by a Typhoon FLA 9000 phosphor imager (GE Healthcare) (**Figure 2**). Proteins within the gel were visualized with Coomassie brilliant blue and GST was used as negative control.

### *In vitro* Kinase–Client assay

Each synthetic peptide was individually resuspended in dimethyl sulfoxide (DMSO, analytical grade ≥99.9%) to a 5 mM concentration. DMSO inhibition of kinase activity had been previously determined to be negligible (**Figure 2**). All samples were kept on ice throughout the resuspension protocol. After solubilization was complete, 10 µL aliquots of each synthetic peptide were transferred to a new Eppendorf to create the 225-peptide pool. The final concentration of each peptide in the 225-peptide pool was 22.2 µM and 10 µL single-use aliquots were prepared and stored at -80°C before use. The 225-peptide library was initially analyzed in its neat form to ensure uniform detection of expected peptides, and later, the *in vitro* KiC assay reaction was performed. Testing of two different buffer types, Tris and HEPES, with purified P2K1-CD, revealed that Tris supported higher phosphorylation efficiency (data not shown). Subsequently, substrate concentration and enzyme-to-substrate ratio were further adjusted to provide optimal phosphorylation efficiency per KiC assay reaction (**Supplementary Table 2**). Thus, a 40 µL KiC assay reaction mixture (final volume) was prepared with kinase buffer (20 mM Tris HCl, pH 7.5, 10 mM MgCl_2_, 5 mM EGTA, 100 mM NaCl, and 1 mM DTT) to ensure optimal enzyme activity. 2 mM ATP was added only to “+ATP” samples. Purified P2K1-CD was quantified by Bradford assay with bovine gamma globulin as standard. The KiC assay reaction was performed at a 1: 1 (enzyme: substrate ratio) concentration by adding 14.72 µg of each CD, reducing peptide usage by 8-fold compared to previous studies (Huang and Thelen, 2012). The 1:1 ratio was chosen as it increased the number of phosphopeptides identified with a consistent overlap among the previously compared ratios (1:4, 1:2, 1:1) (**Supplementary Table 2**). All optimizations were based on 18 samples. Reactions were incubated for 90 min at 30°C -with gentle shaking at 300 rpm. Reactions were quenched by adding an equal volume (40 µL) of 1% formic acid in 99% acetonitrile, which diluted the peptide pool concentration to 0.18 µg µL^-1^ (84.4 µM). Quenched reactions were stored at −20°C. No sample clean-up step was performed before LC-MS/MS analysis. Instead, the usage of a trap column and a longer sample loading step to protect the analytical column were adopted.

### Mass spectrometry analysis

Data acquisition parameters were optimized to maximize peptide coverage and reproducibility among injections. Peptides were separated on a Finnigan Surveyor liquid chromatography (LC) system at 125 μL/min on a split-flow design over a 90-min gradient. Mobile phases consisted of 0.1% formic acid in water (phase A) and 0.1% formic acid in acetonitrile (phase B). Acetonitrile concentration was initially at 5% for 5 min, followed by a constant increase from 5% to 50% phase B over 75 min gradient, gradient to 95% phase B in 5 min, and finally, 5% phase B for 5 min. Data acquisition was performed on an LTQ Orbitrap XL ETD mass spectrometer (Thermo Fisher, San Jose, CA) via data-dependent acquisition (DDA), with the top 11 most abundant ions from the precursor scan selected for subsequent fragmentation. Nanospray ionization source parameter settings were: ion spray voltage (kV), 1.90; capillary temperature (°C), 200; capillary voltage (v), 40; and tube lens (v), 150. Precursor masses were scanned with the analyzer set to FTMS, mass range set to normal, 60000 resolution, positive mode, and centroid data type with a scan range of 200–2000 m/z. Tryptic peptides were fragmented using collision-induced dissociation (CID) with settings as follows: collision energy 35 kV, default charge state +2, isolation width 2.0 m/z, activation time of 30 ms, and minimal signal required of 500. Charge-state screening mode was enabled and unassigned charge states or charge states of +1 were not analyzed. Dynamic exclusion was enabled with a repeat count of 1, repeat duration of 30 s, exclusion list size 100, and exclusion duration of 30 s. 6.94 µL (1.25 µg) of each sample was initially loaded onto a OPTI-TRAP™ cartridge (Optimize Technologies, Oregon City, OR), 5 µL, 0.5mm x 1.3mm, and later, separated over a self-packed Analytical column (20 cm, 75 µm internal diameter, HxSil 5µm C18 matrix).

### Data processing and interpretation

Individual searches of raw files were performed on Proteome Discoverer 2.4 against the 225-peptide FASTA file. Spectra were selected based on their MS1 precursor with minimum and maximum precursor masses of 350 and 5000 Da, respectively. High-confidence identifications were achieved with the Sequest HT search algorithm with a precursor mass tolerance of 10 ppm and a fragment mass tolerance of 0.6 Da. A maximum of four dynamic modifications were allowed for a single peptide, as follows: oxidation of methionine (+15.995 Da), and phosphorylation of S-, T- and Y-residues (+79.966 Da). As a peptide-spectrum match (PSM) validator, Percolator (Käll et al., 2007) was used with an FDR ≤0.01 target for high confidence identifications and ≤0.05 FDR for medium confidence identifications. Precise phosphorylation site determination was achieved with the IMP – ptmRS tool.

Filtration criteria were established based on false-positive identification results from negative-control samples. P2K wild type and dead version CDs were purified and screened against the 225-peptide library under different enzyme: substrate ratios and the presence or absence of ATP (**Supplementary Table 3**). Based upon phosphopeptide identifications across negative control samples, the following criteria were established for valid phosphopeptide identification, and therefore, all phosphopeptide identifications from the WT +ATP condition that did not meet these criteria were excluded from all analyses, as follows: 1) PSM counts ≥ 2; 2) Xcorr > 2.04; and 3) Phosphorylation site successfully elucidated by the IMP-ptmRS tool. Peptide 154 was considered an outlier and excluded from all analyses as its phosphorylated form was identified across all samples, which might be attributed to the peptide’s elevated number of S-residues posing additional challenges for the search algorithm to distinguish between the phosphorylated and non-phosphorylated forms. Results after the final filtration of the P2K1-CD reaction with the 225-peptide library are shown in **Supplementary Table 4**.

Annotated MS/MS spectra for the 46 phosphopeptide candidates are provided in **Supplementary Table 5**. The mass spectrometry proteomics data have been deposited to the ProteomeXchange Consortium via the PRIDE (Perez-Riverol et al., 2022) partner repository with the dataset identifier PXD047713.

### Peptide analysis

Annotation enrichment analysis of GO Biological Process, Molecular Function, and Cellular Component terms among the proteins that contain peptides was performed in the TAIR website (Berardini et al., 2004). Subcellular localization predictions of the detected phosphopeptides were generated through the SUBA4 database, which contains information on both the computationally predicted and experimentally documented subcellular localization of many Arabidopsis proteins (Hooper et al., 2017). In this database, a confidence score for each distinct compartment or region is generated with experimentally determined localizations (either mass-spec or fluorescent protein-based data) weighted five times more than *in silico* predictions. The highest subcellular score for each gene was selected as its final subcellular localization (**Supplementary Table 6**).

For motif analysis, a custom Python script was employed to center-align 225 peptide datasets around their phosphorylation sites, ensuring a uniform representation for downstream analyses. To refine our dataset and eliminate redundancy that might obscure motif detection, we utilized the CD-HIT tool (Fu et al., 2012) with a 90% identity threshold, which clusters sequences based on their similarity and selects a representative peptide from each cluster. This step ensured that the subsequent motif analysis was conducted on a non-redundant set of sequences, enhancing the accuracy and reliability of the motif prediction. After redundancy removal, MEME (Bailey et al., 2009) software was employed to detect motifs among phosphopeptide candidate sequences. The MEME algorithm was configured with an amino acid window ≤5 and <0.05 p-value to identify statistically significant motif patterns. Lastly, phosphopeptide candidates were searched against experimental phosphoproteomic databases such as P3DB (Gao et al., 2009) and PhosPhAt (Heazlewood et al., 2008) using a BLASTP search with an identity threshold of 100% (**Supplementary Table 7**).

## Results

**Figure 3:**
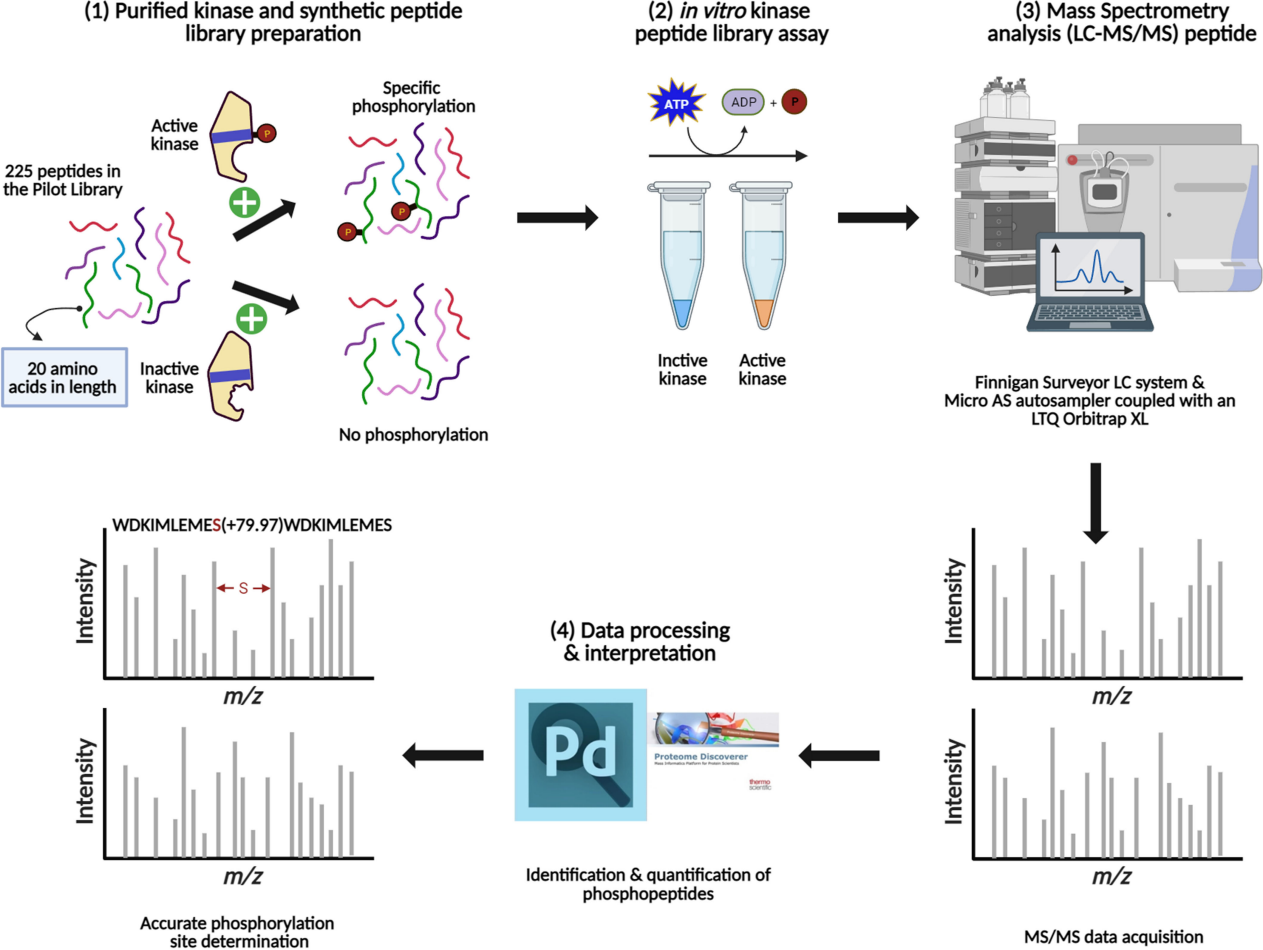
Schematic representation of the main steps involved in the method development for the KiC assay experiment. (1) Kinase purification, activity test, and synthetic peptide library pooling are performed prior to screening. (2) Optimal conditions are provided for *in vitro* KiC assay, which allows phosphorylation events to happen. (3) Samples injection into a liquid chromatography system coupled to a mass spectrometer and MS/MS spectra acquisition. (4) Database search and rigorous filtration are conducted to enable high-confidence phosphorylation and site determination identifications. Figure was created with BioRender.com.

To experimentally test algorithms for predicting protein kinase clients, a 225-member peptide library was designed based upon previous experimentally identified phosphorylated peptides and *in silico* predicted phosphorylated peptides using both MUsite and HMMER tools. All peptides present in the library represent polypeptide sequences of 20 amino acids encoded within the Arabidopsis proteome. The 225-peptide library represents a diversity of biological processes (BP), molecular functions, and cellular components (CC) (**Figure 1**). Salient to signal transduction, “kinase activity” and “nucleus” were the most enriched biological process and cellular component GO-terms. Prior to screening, P2K1-CD phosphorylation activity was confirmed (**Figure 2A**). Confident identification of phosphopeptide candidates with the *in vitro* KiC assay consists of steps: 1) kinase purification and peptide library preparation; 2) *in vitro* screening with specific reagents and concentrations; 3) mass spectrometry data acquisition; and 4) database processing and post analysis to assure high-confidence identifications (**Figure 3**).

**Figure 4:**
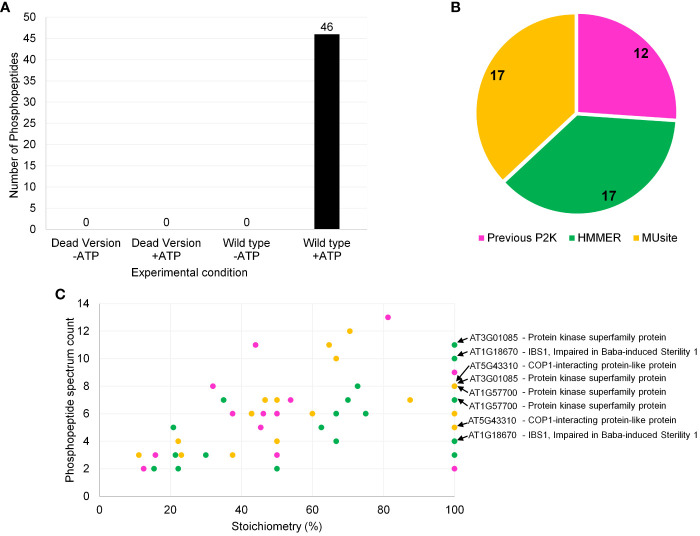
Screening results of P2K1-CD (DORN1) enzyme against a synthetic peptide library containing 225 peptides. **(A)** Number of phosphopeptide identifications for P2K1-CD and GST-p2k1-1-CD across different experimental conditions with quality-control filters. The filtration criteria were as follows: 1) Xcorr > 2.04, 2) PSM counts ≥ 2, and 3) phosphorylation site probability well defined. -ATP and +ATP indicate experimental conditions without and with ATP, respectively. Each bar represents results from a single screening. **(B)** Phosphopeptide identification distribution according to the 225-peptide library sources, which included both *in silico* prediction tools and previous *in vitro* KiC assay results. **(C)** Phosphopeptide classification of P2K1 screening results based on stoichiometry and phosphopeptide spectrum count (Stoichiometry = (phosphopeptide spectrum count)/(total peptide spectrum count) *x* 100). Phosphopeptides with more than one phosphorylation site are also shown in panel **(C)**. Colors from **(B)** and **(C)** panels represent the same peptide origin. Accession numbers of phosphopeptide candidates with the highest confidence identification based on stoichiometry, PSM counts, subcellular localization, and motif analysis are highlighted in panel **(C)**. Repeated accession numbers indicate that the protein is represented by multiple phosphosites in the P2K1 KiC dataset. Additional information can be found in **Supplementary Table 4**.

After reaction condition optimization, which included enzyme-substrate ratio optimization as well as establishing filtration criteria based upon negative controls (**Supplementary Tables 2**, **3**), the results of screening of the P2K1-CD against the 225-peptide library are shown in **Figure 4**. The KiC assay with P2K1-CD enzyme was highly specific, with no background peptides detected above the threshold in negative controls, which included a non-active form of P2K1 (p2k1-1-CD “dead” kinase version) (Choi et al., 2014) under conditions with and without ATP, and the active form of P2K1-CD (Wild type) under conditions without ATP (**Figure 4A**).

P2K1 phosphopeptide candidates included peptides from all three sources: previous experimental result, HMM, and MUsite (**Figure 4B**). Peptides from the two *in silico* prediction approaches were evenly represented among new P2K1 phosphorylated peptides (**Figure 4B**). The even distribution between the two methods suggests that both performed well. High confidence phosphorylation events can be specified by repeated detection of phosphorylated peptides (spectral counts) and the ratio (stoichiometry) of phosphorylated to unphosphorylated peptides detected (**Figure 4C**, **Supplementary Table 4**). Generally, novel phosphopeptide candidates from MUsite and HMM exhibited both higher stoichiometry and spectral counts compared to previously identified phosphopeptides.

**Figure 5:**
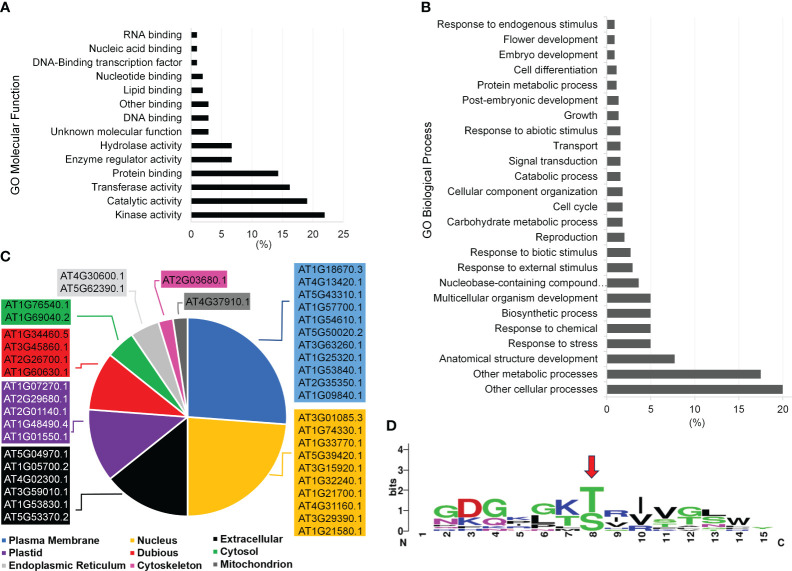
Functional categorization of P2K1 (DORN1) phosphorylated candidates after KiC assay screening with a 225-synthetic peptide library. **(A, B)** Functional categorization by annotation for gene ontology (GO) Molecular Function and GO Biological Process among phosphopeptide candidates, respectively. Only GO classes with >1% abundance among phosphopeptide candidates are shown. **(C)** Subcellular localization of novel clients according to the SUBA4 database. Localizations include predicted and experimental subcellular localizations and the highest-scoring subcellular localization prediction was selected. Complete details of all subcellular localization predictions can be found in the **Supplementary Table 6**. **(D)** Motif analysis among candidate P2K1 peptides by the MEME algorithm at the 0.05 probability level. The red arrow indicates the motif phosphorylation site.

Gene ontology (GO) analysis of the proteins that correspond to the peptides phosphorylated by P2K1-CD might indicate mechanisms by which plants respond to wounding and pathogens. In the category GO-molecular function, the most abundant client candidates showed kinase activity (22%), catalytic activity (19%), transferase activity (17%), and protein binding (14%) (**Figure 5A**). Besides a general GO-biological process term (other cellular processes, 20%), the majority of phosphopeptide candidates were from proteins involved in metabolic processes (17.5%), anatomical structure development (7.7%), and response to stress (5%) and response to chemicals (5%) (**Figure 5B**). These results reinforce that although the number of synthetic peptides present in the library was limited, it is quite diverse covering multiple vital classes of molecular functions and biological processes for plant immunity.

Another important aspect of phosphopeptide candidate characterization is cellular localization. The most abundant terms represented by the protein candidates were plasma membrane (26%) and nucleus (24%) with nearly all localizations being supported with experimentally documented subcellular localization according to SUBA4 database (**Figure 5C**, **Supplementary Table 6**). Given the plasma membrane localization of P2K1 (Choi et al., 2014), phosphopeptide candidates that are localized to either the cytoplasm or plasma membrane would be given higher priority for further *in vivo* confirmatory interaction studies.

We identified a consensus phosphorylation motif among candidate peptides from this experiment (**Figure 5D**). Twelve phosphopeptide candidates were present in this motif, and a significant number also shows 100% stoichiometry as highlighted in **Figure 4C**. Detailed information on all phosphopeptide candidates present among the consensus motif can be found in **Supplementary Table 4**. The consensus motif can provide an extra piece of information on the target phosphopeptide selection together with high stoichiometry identification, which can help elucidate the motif recognition pattern by P2K1 kinase.

After screening against purified P2K1-CD, we identified a total of 46 phosphopeptide candidates. Among them, 19 unique phosphopeptide candidates were found in either P3DB or PhosPhAt phosphoproteome databases (7). The identification of 19 unique phosphopeptide candidates in those phosphoproteome databases serves as additional confirmation of their status as phosphopeptides. Two phosphopeptide candidates identified in the phosphoproteome databases were also associated with the consensus motif generated in the current study (Peptides 31 and 63) (**Supplementary Table 7**). Including peptides that are partially or fully documented in those repositories, 13 candidates were identified in both databases. The remaining 27 phosphopeptide candidates may be novel, as they might not have been identified experimentally elsewhere.

## Discussion

We optimized the KiC assay and obtained results that compared two kinase peptide substrate prediction algorithms while identifying novel putative clients for P2K1. To achieve this, we adopted a strategy that leveraged both experimental data and i*n silico* methodologies. Firstly, the design of the peptide library drew upon prior experimental research that had identified and confirmed a substantial number of phosphorylation events for P2K1 (Chen et al., 2017, 2021; Kim et al., 2023). In addition to experimentally identified peptides, powerful *in silico* prediction tools, such as MUsite and HMMER were employed to determine the efficacy of bioinformatics for phosphorylation prediction. These computational algorithms were employed to explore the vast landscape of potential phosphorylation sites within plant proteomes and to encompass the most relevant and biologically significant phosphorylation sites.

The low background among the negative controls highlights the high specificity of this technique as a tool for discovering and exploring novel substrates for receptor-like kinases. Moreover, an important strategy for enhancing confidence in phosphopeptide candidates is phosphorylation stoichiometry. This quantitative approach considers the number of phosphorylated spectra matches for a particular phosphopeptide in comparison with the total number of peptide spectrum matches, which also includes the non-phosphorylated spectra. Therefore, it can provide additional evidence together with a higher number of PSMs on selecting candidates for further *in vivo* studies. Although experimental validation of clients identified through the KiC assay has been high, further experiments would be needed to determine if the lower confidence peptides are also biologically relevant.

The high rate of phosphorylation on peptides for which no experimental phosphorylation has been detected might indicate that despite decades of research, the experimental plant phosphoproteome remains shallow. The high representation of signaling-related processes for the proteins that contain the detected peptides is consistent with expectations as shown in **Figure 5**. This observation suggests some of the newly identified peptides are biologically relevant. Moreover, the results provide input into development of a bioinformatic pipeline for future prediction of additional kinase substrates based on experimental data that will always be inherently limited. The results of the 225-peptide library analysis with P2K1-CD allow the expansion with a higher number of predicted synthetic peptides based on the current bioinformatic pipelines cited in this work, which has increased the success rate compared to previous experiments (Ahsan et al., 2013). Based on these results, applying *in silico* prediction methods and machine learning algorithms, can ultimately develop a larger library encompassing the most relevant phosphorylation sites.

Many studied RLKs have pivotal functions in cell-cell communication and innate immunity. Notably, previous incarnations of the KiC assay have already contributed valuable insights into the functions of plant P2K RLKs (Chen et al., 2021; Cho et al., 2022). Experimental validation of KiC assay phosphopeptide candidates extends to other kinases. For instance, it was confirmed that the PSY1R receptor interacts with SERK co-receptor family members, undergoes autophosphorylation at specific sites, and that phosphorylation of Ser951 stabilizes the receptor’s inactive conformation (Oehlenschlæger et al., 2017). In another study, SERK1 and SERK2 LRR-RLKs were found to act as coregulators for the EMS1 kinase, enhancing its activity and playing a crucial role in anther development (Li et al., 2017). Additionally, ILK1 kinase was shown to bridge plant defense responses to pathogen-associated molecular patterns (PAMPs) and potassium ion homeostasis, contributing to immunity against bacterial pathogens and being modulated by CML9, a negative regulator of immunity (Brauer et al., 2016). Those confirmatory studies suggest that P2K1 can indeed interact with a variety of substrates and be involved in different biological processes within the plant as also shown in our current data.

In the current study, the percentage of phosphopeptide candidates from HMMER (21%) and MUsite (20%) exceeded the percentage of identified phosphopeptides from a previous *in vitro* screening with a 2k library (2%) (data not shown). One reason for this could be that the synthetic peptides in this 225-peptide library have all been standardized to 20-mers with the phosphosite centered, whereas the previous 2k library was composed of tryptic peptides. The 20-mer peptides might allow for partial secondary structure formation and provide a more relevant binding site for CD-peptide interactions. Another possibility is that the computational methods were able to enrich the library with likely P2K substrates. This result demonstrates the effectiveness of the KiC Assay in identifying direct phosphorylation events, which can later allow the identification of direct interaction partners at the protein level. This comprehensive approach, blending experimental and computational methodologies, underscores the success of prediction algorithms in the KiC assay, paving the way for the discovery of crucial insights into kinase-client interactions.

Beyond stoichiometry and the number of PSMs for high-confidence phosphopeptide candidate identifications, subcellular localization and motif analysis can also provide meaningful insights. Subcellular localization is also an important step for further investigation of *in vivo* interaction experiments. As a member of the lectin-RLK subfamily, P2K1 carries an intracellular kinase domain, a transmembrane domain, and an extracellular lectin domain (Choi et al., 2014). Likewise, we prioritize phosphopeptide candidates for further *in vivo* confirmation that show subcellular localization at the plasma membrane, cytosol, endoplasmic reticulum, and nucleus, respectively. Those localizations are more likely to be associated with P2K1 due to its residence within the plasma membrane. However, it is quickly becoming clear that organelles are intricately interconnected and that these physical relationships at contact locations serve multiple crucial functions (Scorrano et al., 2019), as well as exhibit dual or multiple localizations within subcellular organelles, as previously reported (Hammani et al., 2011; Teardo et al., 2011; Blanco et al., 2019). Therefore, as predictions of subcellular localization rely on both in silico methods and experimental data, whenever available, they should be regarded as tentative results.

A search for phosphorylation motifs in all of the reported phosphopeptide candidates throughout the KiC assay screening with P2K1-CD enzyme was carried out to indirectly infer P2K1 substrate affinity. Based on the phosphopeptide candidates identified in this study, we were able to generate one significant motif that may inform future *in silico* library design and understanding of P2K1 function. Considering the above criteria, two phosphopeptide candidates [Peptides 63 (from AT1G18670) and 65 (from AT1G57700)] stand out as they presented 100% phosphorylation stoichiometry, high number of PSM counts, plasma membrane subcellular localization, and were part of the consensus motif analysis. Interestingly, Peptide 63 was also identified in the database PhosPhAt. On the other hand, two phosphopeptide candidates [Peptides 70 (from AT3G01085) and 150 (from AT5G43310)], were not part of the consensus motif analysis but also showed 100% stoichiometry and were localized in the nucleus and plasma membrane, respectively. Notably, the highlighted phosphopeptide candidates were exclusively identified through the innovative *in silico* prediction tools employed in this study, underscoring the significant potential of this approach.

## Conclusion

This study demonstrates the robustness of the *in vitro* KiC assay approach, which integrated advanced prediction algorithms and efficient selection criteria. Also, it underscores the effectiveness of contemporary prediction algorithms in accurately predicting phosphorylation sites for orphan receptor-like kinases in plants. Utilizing this approach, we successfully identified 46 potential substrates for P2K1, notably uncovering 34 novel phosphopeptide candidates with a high level of confidence, 27 of which may be novel peptides not previously identified experimentally. Our findings provide essential insights regarding selection criteria for subsequent *in vivo* experiments aimed at confirming these discoveries. In doing so, the KiC assay emerges as a pivotal resource, furthering our understanding of the intricate realm of plant phosphorylation and its multifaceted implications in plant biology.

## Data availability statement

The datasets presented in this study can be found in online repositories. The names of the repository/repositories and accession number(s) can be found below: ProteomeXchange, PXD047713.

## Author contributions

GJ: Formal analysis, Investigation, Methodology, Writing – original draft. DK: Formal analysis, Investigation, Writing – original draft. CX: Formal analysis, Writing – review & editing. SC: Formal analysis, Writing – review & editing. LS: Data curation, Writing – review & editing. DX: Conceptualization, Data curation, Funding acquisition, Writing – review & editing. LB: Conceptualization, Data curation, Funding acquisition, Writing – review & editing. GS: Conceptualization, Data curation, Funding acquisition, Writing – review & editing. JT: Conceptualization, Data curation, Funding acquisition, Supervision, Writing – review & editing.

## Supplementary material

Supplementary Table 1 225-peptide library.

Supplementary Table 2 Enzyme: substrate ratio comparison.

Supplementary Table 3 Establishing filtration criteria based upon negative controls.

Supplementary Table 4 Candidates phosphopeptides of P2K1.

Supplementary Table 5 MS/MS spectra of 46 phosphopeptide candidates.

Supplementary Table 6 Subcellular localization predictions.

Supplementary Table 7 Search results of candidates in p3db and phosPhAT databases.
